# Synthesis, Empirical and Theoretical Investigations on New Histaminium Bis(Trioxonitrate) Compound

**DOI:** 10.3390/molecules28041931

**Published:** 2023-02-17

**Authors:** Mahdi Jmai, Sofian Gatfaoui, Noureddine Issaoui, Thierry Roisnel, Aleksandr S. Kazachenko, Omar Al-Dossary, Houda Marouani, Anna S. Kazachenko

**Affiliations:** 1 LR13ES08 Material Chemistry Laboratory, Faculty of Sciences of Bizerte, Université of Carthage, Bizerte 7021, Tunisia; 2 Laboratory of Quantum and Statistical Physics, Faculty of Sciences, University of Monastir, Monastir 5079, Tunisia; 3 CNRS (Centre National de la Recherche Scientifique), ISCR (Institut des Sciences Chimiques de Rennes)—UMR 6226, University of Rennes, 35000 Rennes, France; 4 Department of Organic and Analytical Chemistry, Institute of Non-Ferrous Metals and Materials, Siberian Federal University, Krasnoyarsk 660041, Russia; 5 Krasnoyarsk Scientific Center, Siberian BranchInstitute of Chemistry and Chemical Technology, Russian Academy of Sciences, Krasnoyarsk 660036, Russia; 6 Department of Physics and Astronomy, College of Science, King Saud University, P.O. Box 2455, Riyadh 11451, Saudi Arabia

**Keywords:** organic–inorganic material, histamine, single-crystal X-ray diffraction, infrared spectrum, ultraviolet–visible spectrometry, spectrofluorimetry, DFT calculations

## Abstract

In this paper, a novel hybrid material, entitled histaminium bis(trioxonitrate), with the general chemical formula (C_5_H_11_N_3_)(NO_3_)_2_, denoted by HTN was presented. Single-crystal X-ray diffraction was used to determine the structural characteristics of this compound after it was made using a slow evaporation method at room temperature. This compound was elaborated and crystallized to the monoclinic system with space group *P*2_1_/c, and the lattice parameters obtained were: a = 10.4807 (16)Å, b = 11.8747 (15)Å, c = 16.194 (2)Å, β = 95.095 (6)°, V = 2007.4 (5)Å^3^ and Z = 8. The title compound’s atomic structure couldbe modeled as a three-dimensional network. Organic cations and nitrate anions were connected via N–H...O and C–H...O hydrogen bonds in the HTN structure. The intermolecular interactions responsible for the formation of crystal packing were evaluated using Hirshfeld surfaces and two-dimensional fingerprint plots. The compound’s infrared spectrum, which ranged from 4000 to 400 cm^−1^, confirmed the presence of the principal bands attributed to the internal modes of the organic cation and nitrate anions. Additionally, spectrofluorimetry and the ultraviolet–visible spectrum was used to investigate this compound. DFT calculations were used to evaluate the composition and properties of HTN. The energy gap, chemical reactivity and crystal stability of HTN were quantified by performing HOMO-LUMO frontier orbitals analysis. Topological analysis (AIM), Reduced Density Gradient (RDG), molecular electrostatic potential surface (MEPS) and Mulliken population were processed to determine the types of non-covalent interactions, atomic charges and molecular polarity in detail.

## 1. Introduction

A hybrid material is a system in which both organic and inorganic species coexist. Hybrid materials are very interesting for several reasons: firstly because they combine the properties of the inorganic and organic materials that constitute them, secondly because they can be elaborated under “soft chemistry” conditions and finally because they offer an innovative route to design a wide variety of materials. They do not only represent an alternative creation for academic research, but their improved or unusual functionalities allow the development of innovative industrial applications.

Organic–inorganic hybrid materials have rapidly emerged as a particularly interesting new field of research in materials science, covering many areas of application. We can mention, for example, the use of hybrid materials in catalytic systems [1], optoelectronics [2], and pharmaceutical industry [3].

The fields of medicine are already influenced by the very rapid development and improvement of hybrid materials; these composites are easy for dentists to use because they penetrate easily and quickly into a cavity [4]. These compounds also target the field of cosmetics in particular for applications concerning skin care and protection [5]. Other applications of hybrid materials are emerging in biologic fields; antibody-based affinity biosensors have been used for the detection of various chemical products [6]. The hybridization of materials via bioactive molecules will aid certain cells in their interaction with the surrounding matrix and in their performance of recognition, adhesion, migration, proliferation, and remodeling [7,8]. Hybrid materials are also applicable in the energy field, manufacturing different types of batteries based on hybrid composites [9]. The development of modern hybrid materials also has commercial applications such as in industrial packaging, textile manufacturing (insulated apparel), aerogel products and building installation products [10]. The use of organic–inorganic hybrid materials in automotive coatings is a potentially productive application, not only to provide coloration but also scratch resistance and protection against environmental factors including UV and chemical attack [4]. This variety of applications is the reason for the evolution of publications related to hybrid materials. The performance of a material/system can be described in a 3D space to be designed with a given set of targeted properties. In this work, we are interested in hybrid compounds resulting from the interaction of nitric acid as an inorganic part and histamine as an organic matrix with the aim of combining the properties of the two entities in a single compound. A new phase was prepared and characterized in empirical and theoretical studies.

Nitrate compounds and their structural and vibrational properties are particularly interesting to study from a variety of viewpoints because the coordination modes of these groups have an impact on these compounds’ stereochemistry and reactivity [11,12,13,14].

Histamine is a member of the family of biogenic amines, a substance resulting from metabolism in humans, animals and plants. It is a natural substance present in fish products and their derivatives and in general in all mammals. It is also found in fruits, vegetables and eggs [15]. In humans, the tissues richest in histamine are the liver, the lungs and the skin. Histamine is an important chemical in biochemistry because of its role in these physiological and pathological processes and its use in pharmacology. In addition, histamine is one of the most essential antacoids in the human body, obtained via the enzymatic decarboxylation of histidine [16]; however, the histamine content is relatively low in a healthy person (around 1 μg·L^−1^ of plasma), but very high in an asthmatic person, varying between 10 and 100 μg·L^−1^ of plasma [17]. The crystal structure determination of histaminium bis(trioxonitrate) was undertaken as part of a study concerned with the structures of antihistamine compounds.

## 2. Results and Discussion

### 2.1. Structure Description

**Figure 1:**
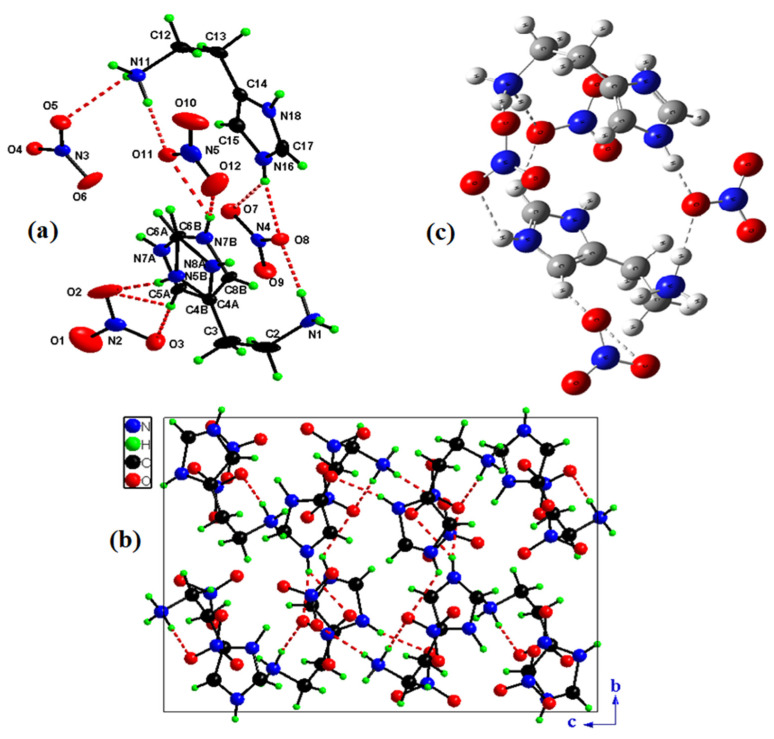
Asymmetric unit of HTN with the atom-labeling scheme (**a**). Projection of HTN structure along the $\vec{a}$ axis (**b**). Optimized structure of (C_5_H_11_N_3_)(NO_3_)_2_ molecule calculated by using B3LYP/ LanL2DZ level (**c**).

Table 1 gives an overview of the crystallographic data and structure refinements. In Figure 1a, the asymmetric unit of the molecular arrangement’s compound HTN can be seen.

The asymmetric unit of the compound (C_5_H_11_N_3_)(NO_3_)_2_ is shown in Figure 1a, formed by four nitrate anions (NO_3_^−^) and two crystallographically independent cations (C_5_H_11_N_3_)^2+^.

The projection of the structure of HTN in the plane ($\vec{b}$, $\vec{c}$) (Figure 1b) shows that organic cations and nitrate anions are connected via N–H...O and C–H...O hydrogen bonds forming a three-dimensional network; we eliminated the disorder only to ensure the clarity of the figure.

This projection shows that the nitrate anions are grouped around a center of inversion of coordinates (1/2,1/2,1/2). Interatomic bond lengths and angles of the nitrate anions spread, respectively, within the ranges 1.218(4)–1.272(3) Å and 118.4(2)–121.9(2)° (Table 2).

**Figure 2:**
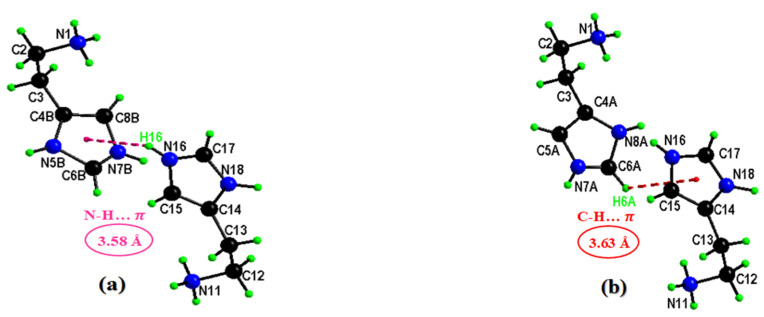
Representation of N–H…π (**a**) and C–H…π (**b**) interactions.

Two crystallographically independent cationic entities are present in the asymmetric unit of HTN with protonations performed on the five nitrogen atoms N11, N18, N1, N8A (0.5 occupancy) and N5B (0.5 occupancy). The different bond angles and interatomic distances of the two histaminium groups are recorded in Table 2. N–H…π interactions (Figure 2a) occur between the organic groups and participate in the stability of the crystal structure with an average distance value equal to 3.58 Å. In addition, C–H…π interactions have been observed in HNA (Figure 2b) with an average distance value equal to 3.63 Å. It is worth noting that these N(C)–H...𝜋 interactions occur between the crystallographically independent organic cations.

The optimized HTN structure given in Figure 1c and Table 2 has been realized with the Gaussian program by using the B3LYP/LanL2DZ level. The variation in the aromatic C–C optimized bond length is 1.385 to 1.386 Å, and the N–C optimized bond length is 1.345 to 1.410 Å. The methyl groups linked to N have the corresponding bond lengths: N1–C5 = 1.514 Å and N20–C24 = 1.5181 Å. Concerning the anionic group the bond length of N–O distributed among the ranges 1.272–1.360 Å, the O–N–O binding angles are calculated between 116.835° and 123.930°. The slight difference in the experimental bond lengths from the optimized bond lengths is explained by the fact that the measurement phase for the experimental results is a crystalline phase, whereas for theoretical results are analyzed in the gas phase.

HTN is very rich in hydrogen bonds (Table 3), and these bonds are subdivided into two types: hydrogen bonds of the N–H...O type connect the protons of the histaminium groups to the oxygen atoms of the nitrate groups with donor-acceptor distances varying from 2.646(6) to 3.255(3) Å, hydrogen bonds of the C–H...O type provide the junction between cations and anions, with donor-acceptor distances varying from 2.716 to 3.298Å, which are relatively long in comparison with those of the N–H...O type. These bonds are considered as weak H-bonds according to the Brown’s criterion (d_C...O_ > 2.7Å) [18].

### 2.2. Hirshfeld Surface Analysis

The presence of hydrogen bonds and intermolecular interactions in the crystal structure of the studied compound was able to be investigated with Hirshfeld surface analysis and was calculated with the Crystal Explorer program [19]. The Hirshfeld surface and its two-dimensional fingerprints [20] have been used to distinguish the various types of interactions such as van der Waals forces, hydrogen bonds, C–H…π and N–H…π interactions.

**Figure 3:**
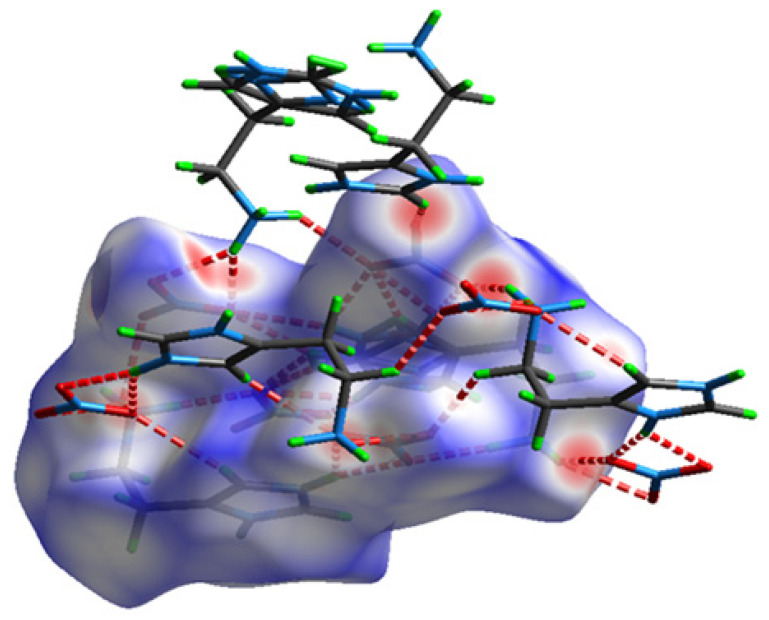
The Hirshfeld surface of HTN mapped in d_norm_ mode (−0.03–1.113).

The Hirshfeld surface of HTN in d_norm_ mode is shown in Figure 3; it is shown in transparent mode to allow the visualization of the molecule inside the surface. The red spots correspond to the close contacts O...H/H...O which are due to hydrogen bonds. The white areas mark the H...H-type contacts on this 3D surface. The blue areas illustrate the domains where the neighboring atoms are too far away to interact with each other.

**Figure 4:**
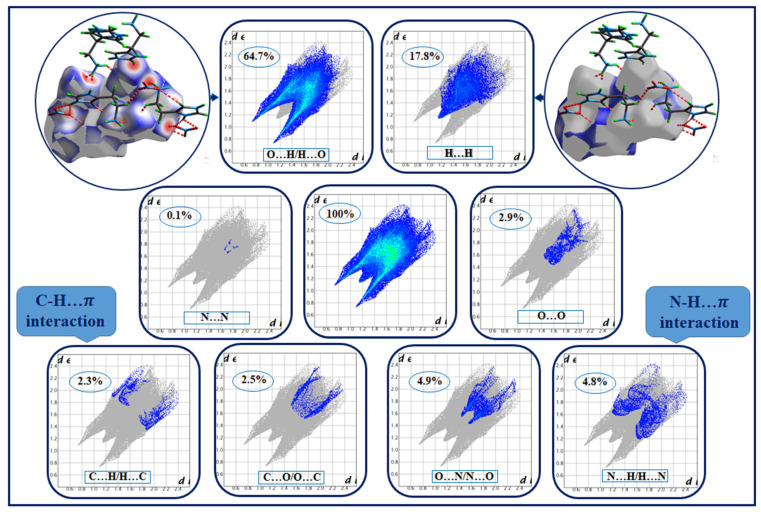
Two-dimensional fingerprint plots of HTN intermolecular contacts.

The 2D fingerprints (Figure 4) show the proportionality giving d_e_ as a function of d_i_; this allowed us to obtain quantitative information on the individual contribution of all interactions in crystalline stacking. This figure shows that the intermolecular O...H/H...O interactions occupy the majority of the total Hirshfeld surfaces (64.7%), which justifies the existence of hydrogen bonds of type N–H....O and C–H...O. Meanwhile, H...H-type contacts are the second most frequent interactions, representing 17.8% of the total surface. We note the presence of H...C/C...H and N...H/H...N-type interactions covering, respectively, 2.3% and 4.8% of the HS, verifying the presence of C–H…π and N–H…π interactions. The other contacts are of type N…O/O…N, O…O, C…O/O…C and N–N, which represent, respectively, 4.9%, 2.9%, 2.5% and 0.1% of the total Hirshfeld surfaces.

### 2.3. Vibrational IR Spectral Analysis

The study of organic substances by spectroscopic methods makes it possible to understand their structural features [21].

**Figure 5:**
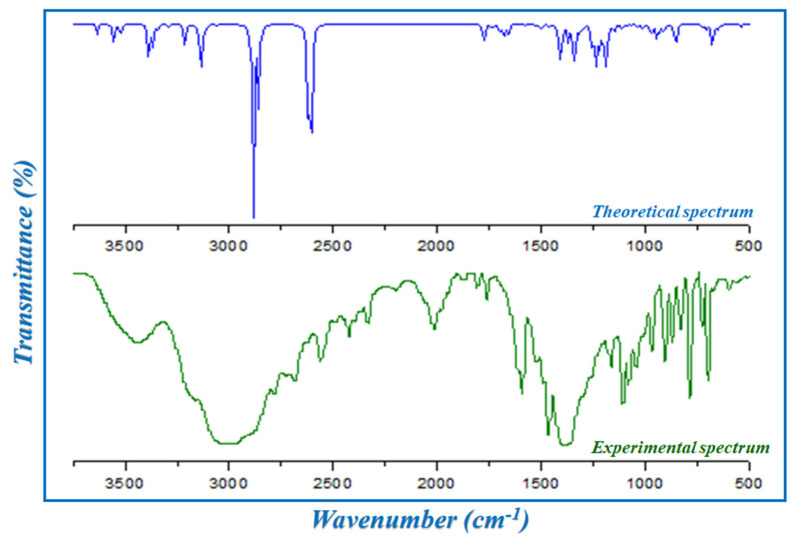
The experimental and theoretical infrared spectra of HTN.

In this section, the different modes of vibration determined during the experimental and theoretical vibrational study of HTN are cited. The infrared spectrum of the synthesized compound is represented in Figure 5 in the 4000–500 cm^−1^ region. The wave numbers of the different atomic groups of HTN are illustrated in Table S1, and based on this table and bibliographic data of the nitrate anion [11,22,23,24] and of the histaminium cation [25], the different modes of vibration of interatomic bonds constituting this crystal can be deduced. The presence of a slight disagreement between the two spectra can be expressed by the phase of measurement for the experimental spectrum, which was a crystalline phase, whereas the theoretical spectrum was analyzed with an isolated molecule and in the gas phase.

#### 2.3.1. Vibration Modes of Histaminium Cation

Concerning the organic entity, the IR absorption spectrum of this compound is characterized by:

A broad band around 3436 cm^−1^ attributed to the valence vibrations υ_s_(N–H) and υ_as_(N–H). The broadening of this band is due to the establishment of H-bonds of type N–H...O. This vibration is calculated between 3632 and 3519 cm^−1^.

The vibration of the CH_2_ groups of the organic cation is observed around 3175 cm^−1^; the calculations give the frequency of this band between 3121 and 3109 cm^−1^.

The second broad band located around 3000 cm^−1^ is associated with the symmetric and asymmetric valence vibrations of the NH_3_^+^ groups; this band is calculated between 2884 and 2857 cm^−1^, which theoretically confirms the vibration of this groups.

A band is experimentally around 1591 cm^−1^,and according to the theoretical calculation, this band is located at 1538 and 1551 cm^−1^, attributed to the asymmetric and symmetric stretching vibration of the CN groups of the organic cation.

#### 2.3.2. Vibration Modes of Nitrate Anion

According to the experimental and theoretical IR spectrum, the NO_3_^−^ anion is characterized by:

A strong band around 1380 cm^−1^ related to the elongation vibration υ(NO_3_^−^); theoretically, this band is calculated around 1346 and 1411 cm^−1^.

Two other characteristic bands of the nitrate anion located around 696 and 1164 cm^−1^ correspond to the deformation vibration β(NO_3_^−^); these bands are interpreted by theoretical calculations, respectively, as being located at 644, 640, 639 and 1189 cm^−1^.

### 2.4. UV-Visible Spectroscopy

**Figure 6:**
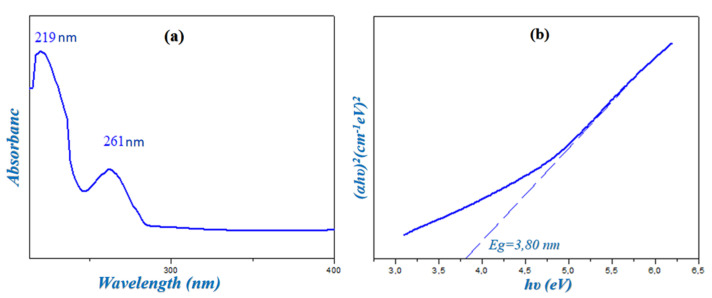
Solution state ultraviolet absorption spectrum of HTN (**a**) and determination of the gap energy obtained via the Tauc model (**b**).

The experimental UV-Visible spectrum of HTN was recorded in water and is represented in Figure 6a; it reveals two characteristic absorption bands: the first band, which is the most intense, located around 219 nm, corresponds to π→π∗ transition of the aromatic cycle of the cationic group; a second band with an absorption maximum located around 261 nm is attributed to the transition *n*→π∗ due to the charge transfer between the nitrate anion and the histaminium cation according to the literature [11]. In the visible region, there is no absorption band, and the compound remains colorless in this region, which justifies the transparent coloring of HTN.

On the other hand, the value of the gap energy was measured by the extrapolation method proposed by Tauc [26] from the variation in(αhν)^2^ as a function of hν, as shown in Figure 6b. This value of E_g_ = 3.80 eV allows this compound to be a semiconductor.

### 2.5. Spectrofluorimetry

**Figure 7:**
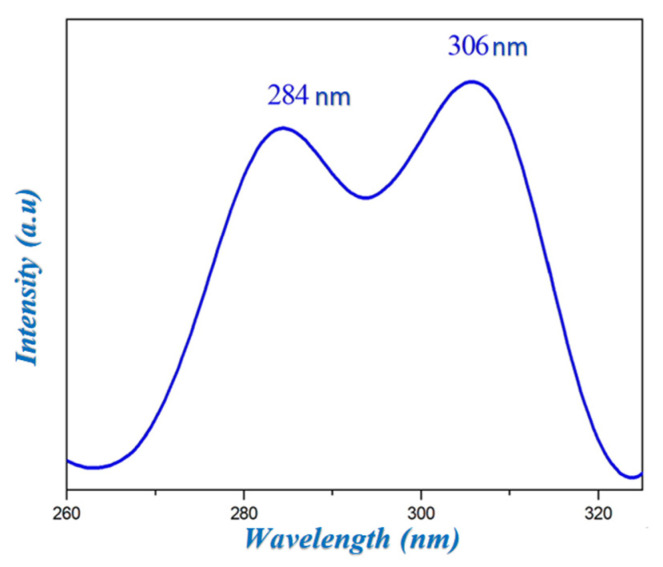
Emission spectrum of HTN.

The luminescence spectrum of HTN is recorded in the solid state at room temperature and represented in Figure 7. This spectrum shows the presence of two strong bands at 284 and 306 nm. These bands can be attributed to the π*→π transitions of the aromatic cycle of the cationic group and π*→*n* related to the charge transfer between the nitrate anion and the histaminium cation.

### 2.6. HOMO-LUMO Analysis

HOMO-LUMO frontier orbitals are two types of molecular orbitals related, respectively, to the nucleophilic and electrophilic character. The HOMO orbital (Highest Occupied Molecular Orbital) is the highest energy molecular orbital occupied by at least one electron and acts as an electron donor; the LUMO orbital (Lowest Unoccupied Molecular Orbital) is the lowest energy orbital not occupied by an electron and acts as an electron hole [27]. The energy gap between the boundary orbitals plays an important role in the electronic properties, molecular reactivity and kinetic stability [28,29].

The calculations were performed using the B3LYP/LanL2DZ method. The energies, electronic affinity (A), global electrophilicity (ω), ionization potential (I), electronegativity (χ), chemical potential (μ), Hardness (ɳ) and Softness (S)are tabulated in Table S2.

The energy values of the HOMO-LUMO molecular orbitals are −6.453472 eV and −2.855728 eV, respectively, and the value of the energy gap obtained is equal to 3.59 eV. The high value of the energy gap obtained will not facilitate the movement of electrons, so the molecule is associated with low chemical reactivity (high kinetic stability). The electrophilicity index measures the stabilization of energy when the system acquires an additional electronic charge, so ahigh value favors its electrophilic behavior [30]. It is worth noting that the chemical potential is negative, which means that our crystal is stable, biologically active and does not spontaneously decompose into its elements [31].

**Figure 8:**
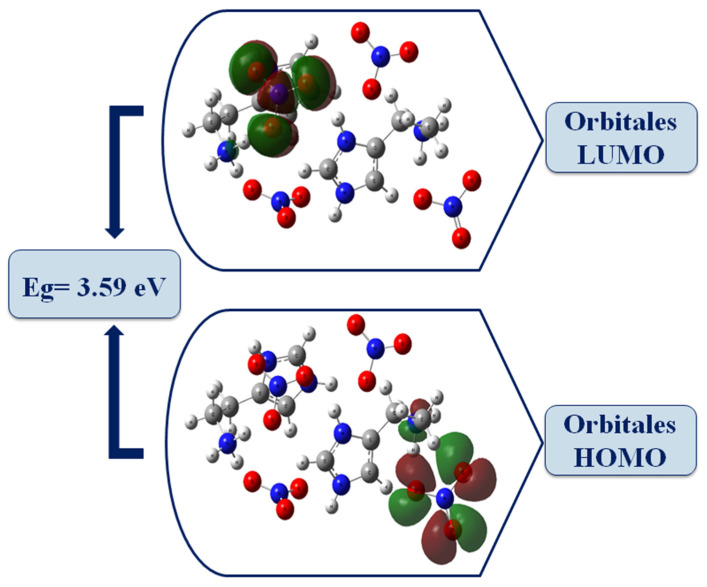
The molecular frontier orbitals of HTN computed using the B3LYP/LanL2DZ level.

Figure 8 gives the HOMO and LUMO representation of the HTN compound; the HOMO orbitals are concentrated on the anionic part and the NH_3_ groups of histaminium, whereas the LUMO orbitals are mainly located on the nitrate anion. It can be deduced that the nitrate anion can act both as an electron donor and acceptor, while histaminium only acts as an electron donor.

The comparison between the gap energy calculated for the HOMO, LUMO molecular boundaries (E_g_ = 3.59 eV) with that evaluated by extrapolation in UV spectroscopy (E_g_ = 3.80 eV) shows that the two methods give close energy values, which means that there is good agreement between the two types of calculations.

### 2.7. AIM Topological Analysis

According to the theory of atoms in molecules (AIM) [32], bond critical points (BCPs) appear when two neighboring atoms are chemically linked or if there is non-bonding interaction between them. AIM has been widely used to determine the types of various binding interactions from the point of view of real space functions such as the electron density at the critical binding points (BCPs). The properties of hydrogen bonds can be determined using topological parameters such as the Laplacian of the electron density ∇^2^ρ(r), electronic density ρ(r), Lagrangian kinetic energy G(r), the energy density potential V(r), Hameltonian kinetic energy H(r) and the energy of interaction E_int_ (kJ·mol^−1^) [33], which are presented in Table 4.

According to Rozas [34], the Laplacian and the Hameltonian kinetic energy H(r) allow one to determine the strength of H-bonds, which can be classified as follows:

− ∇^2^ρ(r) > 0 et H(r) > 0: weak H-bonds.
− ∇^2^ρ(r) > 0 et H(r) < 0: moderate H-bonds.
− ∇^2^ρ(r) < 0 et H(r) < 0: strong H-bonds.

**Figure 9:**
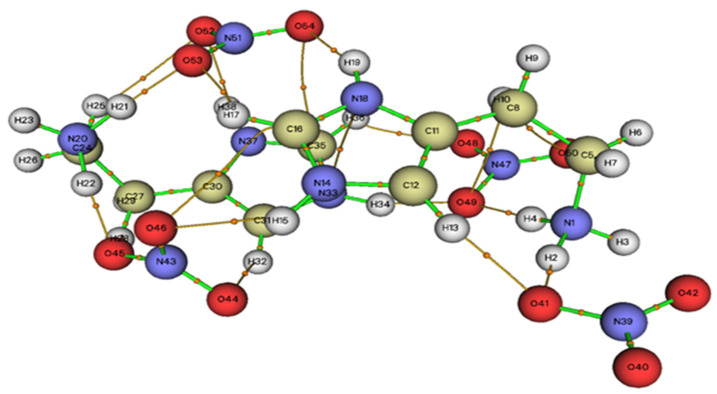
Graphical representation of the AIM analysis of HTN (the critical binding points (BCPs)).

According to the BCP analysis, the HTN compound is stabilized by sixteen interactions divided into eight C–H...O and eight N–H...O, as shown in Figure 9. Five bonds, N1-H4...O49, N33-H34...O49, N1-H2...O41, N20-H22...O45 and N20-H21...O35, have been considered as moderate hydrogen bonds since they have positive Laplacian ∇^2^ρ(r) and negative Hameltonian kinetic energy H(r). The other bonds are considered as weak hydrogen bonds (∇^2^ρ(r) > 0 et H(r) > 0).

### 2.8. Reduced Density Gradient (RDG)Analysis

**Figure 10:**
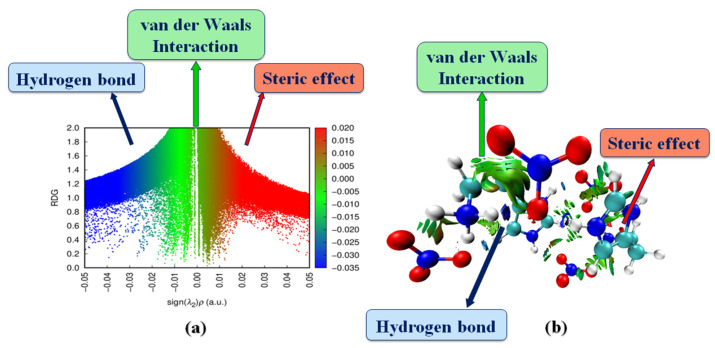
Graphical display of the reduced density gradient scatter (**a**) and iso-surface density (**b**) of HTN to show the non-covalent interactions.

The analysis of the reduced density gradient (RDG) was based on the study of non-covalent interactions (NCIs) existing in our systems [35]. The quantity of electron density as a function of the (sign (λ_2_)*ρ) (Figure 10a) and the iso-surface density (Figure 10b) allowed us to determine the nature and strength of NCI interactions [36].

To characterize intermolecular interactions, we used the signs of λ_2_; they are classified as follows [36]:

− λ_2_ < 0: H-bonding interactions.
− λ_2_ close to zero: van der Waals interactions.
− λ_2_ > 0: steric effect (repulsion; no interaction).

In the RDG graphs, the red color is defined as strong repulsion (steric effect), the blue colors indicate hydrogen bonding interaction and green colors correspond to van der Waals interactions. As shown in Figure 10b, the blue spots between the oxygen atom of the nitrate anion and the hydrogen atoms of the histaminium cation indicate strong interaction corresponding to the N–H...O and C–H...O hydrogen bonds. The van der Waals interaction region can be shown in green, and strong steric effects can be seen in red.

### 2.9. Analysis of the Molecular Electrostatic Potential Surface (MEPS)

The Molecular Electrostatic Potential Surface (MEPS) provides a visual method to understand the relative polarity of compounds [37]; it is mainly used to describe the reactivity related to electrophilic and nucleophilic attacks and in the study of H-bonding interactions [38]. MEPS is a three-dimensional illustration of the charge distribution in a molecule where the electron-rich and partially negative region is shown in red, the area with the highest electrostatic potential is represented by blue, whereas green designates the area of the neutral potential. The potential is increased in an increasing order from positive to negative as follows: blue → green → yellow → orange → red [39].

The MEP surface of HTN calculated using the DFT method is shown in Figure S1. We notice that the region around the oxygen atoms of the nitrate anions represents the most negative potential corresponding to the nucleophilic sites; it is a region with high acceptance of hydrogen bond donor sites. The hydrogen atoms of the histaminium cations having the most positive potential corresponds to the electrophilic sites. These results confirm the charge transfer and the formation of N–H...O and C–H...O hydrogen bonds between the nitrate anions and the histaminium cations, which ensures the crystal stability of HTN.

### 2.10. Mulliken Population Analysis

Many characteristics of molecular structures, including dipole moment, polarizability, and electronic structure, are influenced by the distribution of charge on atoms [40]. Additionally, atomic charges have been used to describe certain chemical reaction processes, such as charge transfer [41].

The calculated Mulliken loads for HTN are shown in Figure S2. From the results obtained, it can be seen that all hydrogen atoms have a positive charge, and the histaminium nitrogen atoms have negative charges because they are attached to positively charged hydrogen atoms. We notice that the N1 and N11 atoms are the most electronegative; this can be explained by the coordination of these atoms with three hydrogen atoms. The nitrogen atoms of the nitrate anion are electropositive because they are surrounded by three negatively charged oxygen atoms. The carbon atoms carrying negative charges, except for C14 and C4A, are electropositive because they are each surrounded by two carbon atoms and one nitrogen atom, which are all negatively charged.

We can deduce that the electronegativity of an element increases by the agreement with another element being positively charged and vice versa.

## 3. Experimental Section

### 3.1. Chemical Preparation

The new organic trioxonitrate (C_5_H_11_N_3_)(NO_3_)_2_ was synthesized via a reaction between nitric acid and histamine. A total of 1 mmol of histamine was dissolved in 15 mL of water; the solution was subjected to magnetic stirring for a few minutes at room temperature. Then, 2 mmol of diluted nitric acid was added drop wise until a pH between 1.5 and 2 was obtained. The solution was then slowly evaporated at room temperature. After a few days, transparent and colorless crystals of prismatic shape and size suitable for structural studies were formed.

### 3.2. Characterization Techniques

#### 3.2.1. Single-Crystal X-ray Diffraction

The instrument used for X-ray diffraction was an APEXII-CCD (Bruker-AXS) diffractometer equipped with a CCD detector, the X-ray source of which has a monochromatic wavelength λ = 0.71073 Å of Molybdenum Kα radiation (Mo Kα). Measurements were performed in a range in θ from 2.5 to 27.5°. Absorption corrections were achieved via the multi-scan technique using the SADABS program [42]. The total number of measured reflections was 15384, among which, 4589 were independent and 3901 had the intensity I > 2σ(I). Direct methods were used to solve the structure using SHELXS-97, which indicated each atom other than a hydrogen atom’s position, and then refined with full-matrix least-square methods based on F^2^(SHELXL-97) [43] included in the WINGX program [42]. R(F^2^) = 0.073 was the result of the final F^2^ refinement.

#### 3.2.2. Physical Measurements

Using the method of pellets with KBr as a dispersant, the IR spectrum was collected at room temperature in the frequency range of 4000–400 cm^−1^.

The ultraviolet spectroscopy in the liquid state was measured at room temperature between 200 and 400 nm using a PerkinElmer Lambda 11 UV/Vis spectrophotometer.

The solid fluorescent spectra were measured at room temperature using a PerkinElmer LS55 spectrofluorometer.

#### 3.2.3. Computational Details

The structure of HTN was modeled using the GaussView program [44], and after that, with the Gaussian 09 software package, this structure was optimized in the gas phase [45]. Using Density Functional Theory (DFT), all quantum chemistry calculations were performed with the aid of the hybrid B3LYP (Becke-3–Lee–Yang–Parr) method and the LanL2DZ (Los Atoms National Laboratory 2-double-zeta) basis set [46,47]. For molecules that exhibit inter or intra-molecular interactions, the B3LYP-D3 functional corrected for dispersion is most preferred [48]. In this paper, we refer to Grimme correction. For long-distance dispersion interactions, Grimme correction was used at D3 [49].

To understand the polarity relative to HTN, the molecular electrostatic potential surface (MEPS) analysis was performed [38]. Charge distribution on atoms, polarizability and electronic structure were evaluated by using the Mulliken population [39]. The theory of atoms in molecules (AIM) was used to determine the types of various interactions in the molecular system [32], specifically to determine the different types of hydrogen bonds [34]. The analysis of non-covalent interactions existing in our systems has been studied using reduced density gradient (RDG) analysis [35]. To better understand the mode of interaction between the different entities and to evaluate the energetic behavior of the studied compound [27], the HOMO-LUMO boundary molecular orbitals analysis was performed.

## 4. Conclusions

The present research focuses on the study of the structure, reactivity and properties of (C_5_H_11_N_3_)(NO_3_)_2_(HTN) based on single-crystal X-ray diffraction, spectroscopic signatures and quantum chemistry calculations. HTN crystallizes in a *P*2_1_/c monoclinic system; the analysis of the Hirshfeld surface allowed us to visualize and analyze the intermolecular interactions. The spectroscopic results determined the characteristic peaks related to HTN and showed the presence of π-π* and *n*-π* transitions. The MEP surface allowed us to determine the polarity of HTN, and Mulliken population analysis was used to determine the charges of the atoms in the crystal system. We quantified the different types of hydrogen bonds using the AIM topological analysis. Different types of non-covalent interactions are presented using Reduced Density Gradient analysis (RDG). To confirm the energetic behavior, chemical reactivity and crystal stability of HTN, we used HOMO-LUMO frontier orbital analysis, which informed us that the most occupied orbitals are located in the inorganic part (NO_3_^−^) and NH_3_ of the organic groups, while the unoccupied ones are located on the nitrate anion. We also noticed good agreement between the gap energy of these orbitals (E_g_ = 3.59 eV) with the gap energy determined by the UV spectrum using the Tauc method (E_g_ = 3.80 eV),which confirms that HTN is a semiconductor.

## Figures and Tables

**Table 1:** Crystal data and experimental parameters used for the intensity data collection strategy and final results of the structure determination of HTN.

CCDC Number	2,236,747
Temperature	150 K
Empirical formula	(C_5_H_11_N_3_)(NO_3_)_2_
Formula weight (g·mol^−1^)	474.37
Crystal size (mm)	0.58 × 0.52 × 0.50
Crystal system	monoclinic
Space group	P2_1_/c
a (Å)	10.4807 (16)
b (Å)	11,8747 (15)
c (Å)	16,194 (2)
β (°)	95,095 (6)
Z	8
V (Å^3^)	2007.4 (5)
F (000)	992
Mo Kα (mm^−1^)	0.14
Reflections collected	15,384
Independent reflections	4589
Reflections with I > 2σ(I)	3901
R_int_	0.049
Absorption correction:	*multi-scan* *T*_min_ = 0.838, *T*_max_ = 0.931
Refined parameters	310
R[F^2^ ˃ 2σ(F^2^)]	0.073
wR(F^2^)	0.186
Goodness-of-fit on F^2^	1.097

**Table 2:** Principal intermolecular distances (Å) and bond angles (°) in HNT via X-ray data (with estimated standard deviation in parentheses) and via theoretical calculations.

Parameters	X-ray	Calculated
Bond length (Å)		
Organic		
N1–C2	1.479(5)	1.5144
C2–C3	1.522(4)	1.5402
C3–C4B	1.469(4)	1.5068
C4B–N5B	1.350(6)	1.4099
C4B–C8B	1.377(7)	1.3862
N5B–C6B	1.500(6)	1.3496
C6B–N7B	1.102(6)	1.3452
N7B–C8B	1.438(7)	1.3982
C3–C4A	1.469(4)	1.5068
C4A–N8A	1.356(6)	1.4099
C4A–C5A	1.403(7)	1.3862
C5A–N7A	1.346(7)	1.3982
N7A–C6A	1.136(6)	1.3452
C6A–N8A	1.520(6)	1.3496
C12–N11	1.492(3)	1.5181
C12–C13	1.522(4)	1.5497
C13–C14	1.489(4)	1.5042
C14–C15	1.359(3)	1.5042
C14–N18	1.382(3)	1.3846
C15–N16	1.381(4)	1.3975
N16–C17	1.326(4)	1.3447
C17–N18	1.317(4)	1.3540
Inorganic		
N2–O1	1.218 (4)	1.2716
N2–O3	1.232 (3)	1.3078
N2–O2	1.238 (3)	1.3576
N3–O6	1.241 (3)	1.2880
N3–O5	1.246 (3)	1.3065
N3–O4	1.267 (3)	1.3327
N4–O9	1.240 (3)	1.2847
N4–O7	1.247 (3)	1.2883
N4–O8	1.269 (3)	1.3598
N5–O10	1.227 (4)	1.2961
N5–O12	1.254 (4)	1.2992
N5–O11	1.272 (3)	1.3299
Bond angle (°)		
Organic		
N1–C2–C3	112.7(3)	113.5539
C4B–C3–C2	113.16(2)	116.4708
N5B–C4B–C8B	110.7(4)	105.9464
N5B–C4B–C3	132.7(4)	121.0151
C8B–C4B–C3	116.5(4)	133.0338
C4B–N5B–C6B	101.1(4)	109.6545
N7B–C6B–N5B	112.1(4)	107.7707
C6B–N7B–C8B	113.6(5)	109.8616
C4B–C8B–N7B	102.5(5)	106.7661
C4A–C3–C2	113.6(2)	116.4708
N8A–C4A–C5A	107.9(4)	105.9464
N8A–C4A–C3	142.3(4)	121.0151
C5A–C4A–C3	109.7(3)	133.0338
N7A–C5A–C4A	107.7(5)	106.7661
C6A–N7A–C5A	111.3(5)	109.8616
N7A–C6A–N8A	113.3(4)	107.7707
C4A–N8A–C6A	99.8(4)	109.6545
Inorganic		
O1–N2–O3	121.1 (3)	123.5990
O1–N2–O2	119.4 (3)	116.8351
O3–N2–O2	119.5 (3)	119.5647
O6–N3–O5	121.9 (2)	121.8126
O6–N3–O4	118.4 (2)	118.7139
O5–N3–O4	119.7 (2)	119.4460
O9–N4–O7	121.0 (2)	123.9298
O9–N4–O8	120.0 (2)	118.2186
O7–N4–O8	119.0 (2)	117.8391
O10–N5–O12	120.8 (3)	122.0878
O10–N5–O11	120.4 (3)	119.2345
O12–N5–O11	118.8 (3)	118.6568

**Table 3:** Geometry of hydrogen bonds (Å, °) in HTN.

D–H…A	D–H (Å)	H…A (Å)	D…A (Å)	D–H…A (°)
N1–H1A···O4 ^i^	0.86(4)	2.20(4)	2.951(3)	146(3)
N1–H1A···O6 ^i^	0.86(4)	2.38(4)	3.101(4)	141(3)
N1–H1B···O12 ^ii^	0.92(4)	2.19(4)	2.978(4)	144(3)
N1–H1B···O2 ^i^	0.92(4)	2.25(4)	2.885(4)	126(3)
N1–H1C···O8	0.94(4)	1.95(4)	2.881(3)	170(3)
N5B–H5B···O2	0.88	2.13	3.001(7)	169
N7B–H7B···O12	0.88	1.79	2.646(6)	164.2
N7B–H7B···O11	0.88	2.30	2.897(5)	124.9
N11–H11A···O11	0.97(3)	1.87(3)	2.835(3)	178(3)
N11–H11B···O5	0.87(3)	2.34(3)	2.809(3)	114(3)
N11–H11B···O7 ^iii^	0.87(3)	2.39(4)	3.212(3)	156(3)
N11–H11B···O9 ^iii^	0.87(3)	2.53(3)	3.255(3)	142(3)
N11–H11C···O3 ^iv^	0.87(4)	2.12(4)	2.902(3)	149(3)
N16–H16···O8	0.82(4)	2.04(4)	2.843(3)	168(3)
N16–H16···O7	0.82(4)	2.51(4)	3.157(3)	136(3)
N18–H18···O4 ^v^	1.04(4)	1.80(4)	2.821(3)	167(3)
N18–H18···O5 ^v^	1.04(4)	2.23(4)	2.993(3)	129(3)
C2–H2A···O10 ^vi^	0.99	2.47	3.214(4)	131.8
C3–H3B···O12 ^ii^	0.99	2.53	3.298(5)	134.4
C5A–H5A···O2	0.95	2.14	2.716(8)	117.9
C5A–H5A···O3	0.95	1.90	2.849(7)	177.9
C12–H12B···O9 ^iv^	0.99	2.55	3.276(3)	130.2

Symmetry codes: (i) x, −y + 1/2, z + 1/2; (ii) −x, y − 1/2, −z + 3/2; (iii) −x + 1, −y + 1, −z + 1; (iv) x, y + 1, z; (v) x, −y + 3/2, z + 1/2; (vi) x, y − 1, z.

**Table 4:** Topological parameters of HTN.

	ρ(r)	$\nabla^{2}\rho (r)$	V(r)	G(r)	H(r)	(Eint) kJ·mol^−1^
C8–H10…O49	0.6060935637	0.2429405290	−0.3508914227	0.4791213726	0.1282299500	0.4552689789
C8–H10…O50	0.5767933635	0.2356048186	−0.3274099481	0.4582109973	0.1308010491	0.4816467982
C12–H13…O41	0.2484511638	0.1052256607	−0.2249624021	0.2440132769	0.1905087482	0.9567061030
C16–H17…O53	0.1559634154	0.7150801709	−0.1170034581	0.1478867504	0.3088329231	0.6862805495
C27–H28…O45	0.1130722919	0.5092741191	−0.7359125289	0.1004548913	0.2686363845	0.4567065471
C31–H32…O44	0.1108296139	0.4708767916	−0.7156373759	0.9464146774	0.2307773015	0.4925806261
C35–H36…O48	0.1354238925	0.6309719703	−0.9453516802	0.1261390803	0.3160391227	0.5748029379
C24–H25…O52	0.5940881497	0.2260591172	−0.2941928340	0.4296703135	0.1354774795	0.5076018638
N1–H2…O41	0.6472117739	0.1796462357	−0.6363728811	0.5427442351	−0.9362864596	0.3174672431
N14–H15…O46	0.1742905363	0.7616909236	−0.1319345448	0.1611786379	0.2924409302	0.1015512073
N20–H22…O45	0.6370579352	0.1861499875	−0.6422615363	0.5538182526	−0.8844328377	0.3685782364
N20–H21…O35	0.5414942347	0.1750245594	−0.5291885920	0.4833749953	−0.4581359667	0.3247555529
N18–H19…O54	0.2685397276	0.1003081517	−0.2228771104	0.2368237449	0.1394663448	0.1784953431
N37–H38…O52	0.1437474207	0.6870277662	−01021190421	0.1369379918	0.3481894974	0.1017548931
N1–H4…O49	0.3973762041	0.1368061188	−0.3618440937	0.3519296954	−0.9914398322	0.2094545303
N33–H34…O49	0.5365923450	0.1691408063	−0.5113382011	0.4670951085	−0.4424309259	0.3126712014

## Data Availability

Not applicable.

## Supplementary Materials

The following supporting information can be downloaded at: https://www.mdpi.com/article/10.3390/molecules28041931/s1, Figure S1: The representation of the Mulliken charge populations calculated for the HTN; Figure S2: The total electron densities mapped with the potential electrostatic surface of the HTN; Table S1: Assignment of experimental and theoretical wave numbers (cm^−1^) of different atomic groups of HTN; Table S2: Parameters of the HOMO-LUMO boundary molecular orbitals of HTN.
