# Supplementary material for: Synthesis, Empirical and Theoretical Investigations on New Histaminium Bis(Trioxonitrate) Compound
Source: Molecules. 2023 Feb 17;28(4):1931. doi: 10.3390/molecules28041931 (PMC9958629; doi:10.3390/molecules28041931)
Supplement: Supplementary file 1 [file molecules-28-01931-s001.zip › molecules-2228896-supplementary.pdf]

Synthesis, Empirical and Theoretical Investigations on New Histaminium Bis(Trioxonitrate) Compound

Mahdi Jmai [1], Sofian Gatfaoui [1], Noureddine Issaoui [2,*], Thierry Roisnel [3], Aleksandr S. Kazachenko [4,5,*], Omar Al-Dossary [6], Houda Marouani [1,*] and Anna S. Kazachenko [4]

[1] LR13ES08 Material Chemistry Laboratory, Faculty of Sciences of Bizerte, Université of Carthage, Bizerte7021, Tunisia
[2] Laboratory of Quantum and Statistical Physics,Faculty of Sciences, University of Monastir, Monastir 5079, Tunisia
[3] CNRS (Centre National de la Recherche Scientifique), ISCR (Institut des Sciences Chimiques de Rennes)–UMR 6226, University of Rennes, 35000 Rennes
[4] Department of organic and analytical chemistry, Institute of Non-Ferrous Metals and Materials, Siberian Federal University, Krasnoyarsk 660041
[5] Krasnoyarsk Scientific Center, Siberian BranchInstitute of Chemistry and Chemical Technology, Russian Academy of Sciences
[6] Department of Physics and Astronomy, College of Science, King Saud University, BO Box 2455, Riyadh 11451, Saudi Arabia
* Correspondence: issaoui_noureddine@yahoo.fr (N.I.); leo_lion_leo@mail.ru (A.S.K.); houdamarouani2015@gmail.com (H.M.)

Supplementary Materials

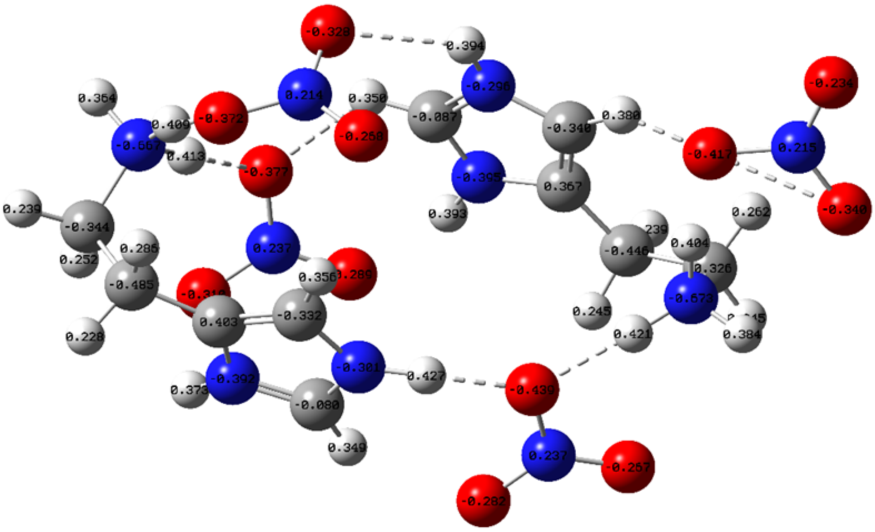

Figure S1. The representation of the Mulliken charges populations calculated for the HTN.

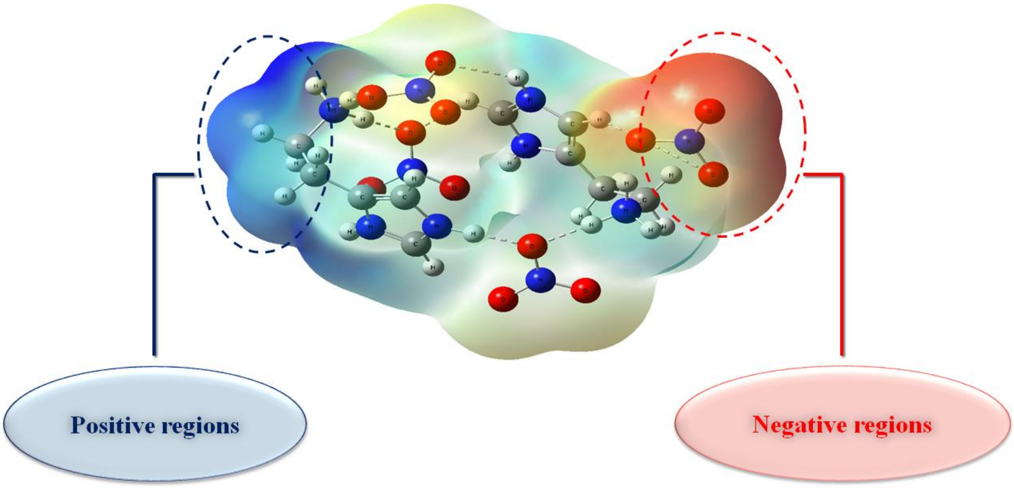

Figure S2. The total electron densities mapped with the potential electrostatic surface of the HTN.

Table S1. Assignment of experimental and theoretical wave numbers (cm^{-1}) of different atomic groups of HTN

Experimental IR	Calculated frequencies	Vibrational assignments (% PED)	Experimental IR	Calculated frequencies	Vibrational assignments (% PED)
3436 m, L	3632	υNH (99)	970 m	976	υON (85)
3436 m, L	3555	υNH (99)	970 m	973	υON (76)
3436 m , L	3523	υNH (99)	-	968	δHCCC (44)
3436 m, L	3519	υNH (99)	-	950	υON (83)
-	3387	υCH (69)	-	947	υON (70)
-	3387	υNH (29)	-	1496	βNCN (10)
-	3346	υCH (98)	-	928	βCNC (29)
-	3293	υCH (99)	-	928	HCCC (12)
-	3214	υCH (98)	-	1307	βHCC (10)
-	3183	υCH (98)	-	1042	δHCCC (12)
-	3164	υCH (92)	-	1012	δHCNC (14)
-	3147	υCH (82)	-	1409	δHCCC (11)
-	3138	υNH (82)	-	916	υNC (10)
3175 m	3121	υCH (81)	905 m	907	δHNCC (13)
3175 m	3109	υCH (83)	1030 m	1032	υNC (12)
3175 m	3098	υCH (97)	780 F	727	δOOON (95)
3175 m	3147	υCH (10)	780 F	723	δOOON (98)
3175 m	3109	υCH (10)	780 F	719	δOOON (97)
3000 F, L	2884	υNH (89)	780 F	713	δOOON (96)
3000 F, L	2857	υNH (92)	-	706	δCCNC (21)
2680 m	2619	υNH (90)	-	706	δHNCC (32)
2680 m	2605	υNH (89)	-	665	δCCNC (53)
1756 f	1777	βHNH (64)	-	664	βONO (61)
1756 f	1771	βHNH (53)	-	651	υCC (10)
1756 f	1738	βHNH (66)	-	654	βONO 60)
1756 f	1707	βHNH (44)	-	656	βONO (19)
1756 f	1693	βHNH (63)	-	656	βONO (10)
-	1777	δHNHO (10)	-	656	δCNCN (19)
	1663	υCC (49)	-	928	δCNCN (10)
-	1653	υCC (48)	1164 M	1189	βONO (10)
-	1663	βHNC (12)	696 F	644	βONO (13)
1591 F	1551	υNC (57)	696 F	640	βONO (71)
1591 F	1538	υNC (57)	696 F	639	βONO (65)
-	1530	βHCH (69)	-	624	υCC (22)
-	1526	βHCH (77)	596 f	543	βNHO (47)
-	1513	βHCH (71)	596 f	538	βNHO (16)
-	1508	βHCH (81)	-	493	βCCN (45)

-	1653	βHNC (16)	-	475	βCCN (14)
1464 F	1464	βHNC (55)	-	380	βCCC (52)
1464 F	1663	βHNC (11)	-	475	βCCC (18)
-	1530	δHCCC (23)	-	315	βCCC (17)
-	1526	δHCCC (16)	-	304	υOH (44)
-	1513	δHCCN 10)	-	282	υOH (42)
-	1418	δHCCN (27)	-	272	δCCCN (49)
1380 F, L	1411	υON (43)	-	315	υOH (23)
970 m	928	δHCCC (15)	-	218	υOH (59)
-	1402	υON (56)	-	202	βCCC (29)
-	1441	βHCN (12)	-	65	δHNCC (14)
-	1362	βCCN (11)	-	171	υOH
-	1346	βHCN (15)	-	543	δNHON (11)
1380 F, L	1346	υON (16)	-	282	δNHON (10)
1380 F, L	1346	υON (18)	-	155	δCNHO (10)
-	1318	βHCC (45)	-	155	δHOHN (10)
1306 m	1307	HCCN (20)	-	143	βNHO (15)
-	1460	υNC (10)	-	1693	δHNHO (16)
-	1259	βCCN (11)	-	133	δHOHN (11)
-	1259	υON (20)	-	121	βHON (39)
-	678	δCCNC (19)	-	114	δHNHO (31)
1247 m	1238	υON (64)	-	110	γCCCC (11)
1247 m	1222	υON (54)	-	104	δCCCN (24)
-	1496	υNC (14)	1756 f	1777	βHNC (10)
-	1679	δCNHO (15)	-	102	βHON (12)
-	1418	βHCC (13)	-	39	δCCCN (11)
-	1738	δHNHO (13)	-	81	δCHON (20)
1164 m	1189	υON (54)	-	1149	βNHO (13)
-	1418	βHCC (10)	-	195	δCCCN (11)
-	1551	βHCN (20)	-	73	δHONO (11)
-	1149	δHNCN (44)	-	165	δHNHO (33)
1105 F	1297	υNC (12)	-	79	δHONO (16)
1105 F	1111	υNC (43)	-	141	βHON (12)
1105 F	1088	υNC (68)	-	45	δHNHO (11)
1080 m	1065	υCC (76)	-	59	δHONO (11)
-	1318	δHCCN (11)	-	45	δHNCN (10)
-	1538	βHCN (21)	-	202	δHNCC (16)
-	1012	δHNCN (37)	-	34	βHOH (13)
-	1006	βNCN (54)	-	155	δHONO (17)
-	987	βCNC (34)	-	45	βHON (19)

Abbreviations: υ, stretching; β, bending in plane; δ, torsion; γ, bending out of the plane, PED, potential energy distribution data.

Table S2. Parameters of the HOMO-LUMO boundary molecular orbitals of HTN

E_{HOMO} (eV)	-6.453472
E_{LUMO} (eV)	-2855728
$\lvert E_{HOMO} - E_{LUMO}\rvert$ Gap (eV)	3.59
E_{HOMO-1} (eV)	-7.22704
E_{LUMO+1} (eV)	-2.275552
$\lvert E_{HOMO-1} - E_{LUMO+1}\rvert$ Gap (eV)	4.951488
Ionization potential: I= $-E_{HOMO}$	6.453472
Electronic affinity: A= $-E_{LUMO}$	2.855728
Electronegativity : χ= (I + A) /2	4.6546
Chemical potential: μ=-(I + A)/2	-4.6546
Hardness : η= (I–A)/2	1.798872
Softness : S=1/2η	0.277952
Global electrophilicity: ω= μ^2 /2η	6.021913
